# Retrospective Study of T Cell Leukaemia (Large Granular Lymphocyte Variant) in Dogs Associated with Suspected Immune-Mediated Cytopaenia(s) in the Absence of Peripheral Lymphocytosis

**DOI:** 10.3390/ani13030357

**Published:** 2023-01-20

**Authors:** Angelo Capasso, Elizabeth Villers, James Elliott, Nic Ilchyshyn, Ian Hopkins, Ferran Valls Sanchez, Sara Verganti

**Affiliations:** 1 Department of Oncology, Dick White Referrals, Station Farm, London Road, Six Mile Bottom CB8 0UH, UK; 2 Department of Pathology, Dick White Referrals, Station Farm, London Road, Six Mile Bottom CB8 0UH, UK; 3 Department of Oncology, Southfields, Cranes Point, Gardiners Ln S, Basildon SS14 3AP, UK; 4 Oackwood Veterinary Referrals, Willows Veterinary Hospital, Chester Road, Hartford, Nortwich CW8 1LP, UK; 5 Department of Internal Medicine, Dick White Referrals, Station Farm, London Road, Six Mile Bottom CB8 0UH, UK

**Keywords:** large granular lymphocyte leukaemia, immune-mediated neutropaenia, cytopaenias, dog, chronic lymphocytic leukaemia, bone marrow, chemotherapy, T cell leukaemia

## Abstract

**Simple Summary:**

Large granular lymphocyte leukaemia associated with severe neutropaenia is extremely rare in dogs. A search of the veterinary literature identified only two case reports. The present study included six dogs with this condition. All patients presented with pyrexia and lethargy and had severe neutropaenia on haematology (median neutrophil count 0.07 × 10^9^/L) with no peripheral lymphocytosis. In all dogs, bone marrow cytology revealed infiltration of granular T lymphocytes. All patients received systemic chemotherapy (five received chlorambucil and prednisolone, one received vincristine, cyclophosphamide, and prednisolone); resolution of the neutropaenia was observed within 19 weeks. Two dogs were euthanised 133 and 322 days after diagnosis due to progressive disease, two were lost to follow-up after 224 and 357 days, and two were alive and disease free at 679 and 721 days. The aim of this retrospective study was to describe the clinical characteristics, treatments, and outcomes of dogs with chronic LGL leukaemia with suspected immune-mediated cytopaenia(s) upon presentation. This condition should be considered in the differential diagnosis of patients presenting with severe neutropaenia.

**Abstract:**

Canine chronic large granular lymphocyte (LGL) leukaemia is commonly characterised by moderate to marked lymphocytosis but not neutropaenia. In humans, LGL leukaemia is often associated with autoimmune disorders, including immune-mediated cytopaenias (mainly neutropaenia). This presentation is rare in dogs. The aim of this retrospective study was to describe the clinical characteristics, treatments, and outcomes of dogs with chronic LGL leukaemia with suspected immune-mediated cytopaenia. Six dogs with a median age of 4.5 years (range 2–8 years) were included in the study. The most common presenting signs were pyrexia and lethargy. All dogs had severe neutropaenia (median neutrophil count 0.07 × 10^9^/L), three had thrombocytopaenia (median platelet count 66 × 10^9^/L), and one had anaemia (HCT 0.32 L/L). In all dogs, bone marrow cytology revealed infiltration of granular T lymphocytes; PARR analysis confirmed clonality in four, and bone marrow flow cytometry identified CD3+ CD8+ neoplastic cells in two cases. All patients received systemic chemotherapy, and the cytopaenias resolved after 1–19 weeks. Two dogs were euthanised 133 and 322 days after diagnosis, two were lost to follow-up after 224 and 357 days, and two were alive at 546 and 721 days. A subset of LGL leukaemia in dogs is associated with immune-mediated cytopaenia and has a unique clinical presentation.

## 1. Introduction

In humans, there are three categories of large granular lymphocyte (LGL) leukaemia (LGLL): chronic T cell leukaemia and NK cell lymphocytosis, which are both indolent diseases characterised by cytopaenias and autoimmune conditions, and aggressive NK cell LGL leukaemia [1]. In humans with LGLL, lymphocytosis is reported in less than half of the patients, with lymphocyte counts ranging from 4.0 to 10.0 × 10^9^/L, and an average of 1.7 × 10^9^/L [1]. The most important haematological finding is neutropaenia, which is seen in 80% of cases, with 17–24% being reported as severe [2,3,4]. While many patients have positive antinuclear antibodies and rheumatoid factor, the pathogenesis of LGL-associated neutropaenia is not fully understood. However, it is likely to be multifactorial; humoral abnormalities, bone marrow infiltration/substitution, and cell-mediated cytotoxicity are likely to be involved [2,5]. Due to the rarity of this condition, with an incidence in humans of 0.2 and 0.72 cases per million per year based on American and Dutch registries, respectively [2], no standard treatment protocol exists. Patients that present with immune-mediated neutropaenia are treated with different immunosuppressive medications, such as cyclosporine or cyclophosphamide, along with granulocyte colony-stimulating factor [6].

In dogs, chronic LGL lymphocytic leukaemia is typically characterised by marked circulating lymphocytosis in the absence of severe cytopaenias. It affects older dogs, with a median age at diagnosis of 10.5 years [7,8]. Only two cases of LGL leukaemia with associated immune-mediated cytopaenia have been reported in veterinary medicine [9,10], and treatment has been described in only one case [10]. In contrast to the more common form, this variant presents with an absence of circulating lymphocytosis but with one or more cytopaenia(s). Rarely, a few LGLs in the peripheral blood have been detected [7,9,11].

In both humans and dogs, when treatment fails, uncontrolled secondary infections caused by severe neutropaenia or haemorrhage due to thrombocytopaenia are common causes of death [10,12,13].

The aim of this retrospective descriptive study was to describe the clinical presentations, laboratory findings, and outcomes of dogs affected by this condition and to assess possible similarities with their human counterparts.

## 2. Materials and Methods

The laboratory databases of the authors’ affiliations were searched to select dogs affected by this lymphoproliferative disorder from 2010 to 2020. The inclusion criteria consisted of a diagnosis of chronic large granular lymphocyte T cell leukaemia (LGL T cell CLL) identified in the bone marrow with a combination of microscopy, clonality testing, and/or flow cytometry, together with associated single or multiple cytopaenia(s) in the absence of circulating lymphocytosis. Eight cases were initially identified, but clinical information was available for only six patients. One of the cases included in our study has been published as a case report [10].

## 3. Results

### 3.1. Clinical Evaluation and Findings

Of the six dogs included, two were Golden Retrievers, and there was one of each of the following: miniature Dachshund, Jack Russell Terrier, Labrador Retriever, and a cross breed. There were three males (one entire and two neutered) and three females (one entire and two neutered). The median age upon presentation was 4.5 years (range 2–8 years). Pyrexia (50%) and lethargy (66%) were common clinical signs at the initial presentation. One dog (16%) had multiple carpal and tarsal joint effusions, and one dog presented with dysphagia. The clinical signs were periacute in all cases.

The investigations performed in each case varied and were clinician dependent. Haematology (ADVIA 2120, Siemens, Camberley, UK) and biochemistry (Olympus AU480, Beckman Coulter, High Wycombe, UK) were performed in all cases. Severe neutropaenia was detected in all cases, with a neutrophil count ranging from 0.02 to 0.76 × 10^9^/L (reference interval (RI): 3.0–11.5 × 10^9^/L). There was no evidence of toxic changes upon evaluation of the blood smear. Thrombocytopaenia was observed in three patients, with a range from 50 to 102 × 10^9^/L (RI: 200–500 × 10^9^/L). Mild nonregenerative normocytic normochromic anaemia was identified in one case (0.32 L/L, RI: 0.37–0.55 L/L). None of the dogs had peripheral lymphocytosis, although one case had an increase in the number of circulating large granular lymphocytes with a normal peripheral lymphocyte count (2.47 × 10^9^/L, RI: 1.2–4.7 × 10^9^/L). In two dogs, moderate lymphopaenia was observed (ranging from 0.11 to 0.6 × 10^9^/L, RI: 1.0–4.8 × 10^9^/L) (Table 1). Biochemistry was normal in all cases. Full urinalysis performed in five dogs and urine culture performed in four dogs were negative for inflammation or infection at the time of presentation, with no abnormalities detected.

All patients also had abdominal and thoracic imaging (3/6 had thoracic radiographs and abdominal ultrasound, 3/6 had thoracic and abdominal CT scans), which did not show any intrathoracic or intra-abdominal abnormalities, including the absence of lymphadenomegaly. Sampling of the liver and spleen was performed in only one dog despite the normal appearance of these organs on ultrasound; it showed normal splenic and hepatic tissue. However, a small number of LGL lymphocytes were detected in the splenic sample, which could reflect splenic involvement or only peripheral blood contamination.

Four patients were also tested for infectious disease, one with PCR on EDTA blood for *Leishmania*, *Babesia*, *Borrelia*, *Ehrlichia*, and *Anaplasma*, and three with serology for *Ehrlichia canis/ewingi*, *Anaplasma phagocytophilum/platys*, *Dirofilaria immitis*, and *Borrelia burgdorferi* (IDEXX 4DX snap test, IDEEX Laboratories, Westbrook, Main, USA); all results were negative. One patient had rheumatoid factor -RF- (IDEXX Laboratories, Wetherby, UK) and antinuclear antibody -ANA- (IDEXX Laboratories, Wetherby, UK) tests performed, which were negative.

In the case presenting with multiple joint effusions, arthrocentesis of the carpal and tarsal joints was performed, and the samples were submitted for cytology and culture. Cytology of the synovial fluid showed increased cell counts, with increased numbers of lymphocytes accounting for 20–30% of the total cells and many activated large mononuclear cells (synoviocytes or macrophages), indicating reactive change. The culture was negative.

The one patient presenting with dysphagia had a history of chronic tonsillitis. Biopsy of the tonsils was performed, and histopathology revealed bilateral chronic neutrophilic and lymphoplasmacytic inflammation.

Bone marrow cytology (Modified Wright stain, Siemen’s Healthcare Diagnostics Manufacturing Ltd., Dublin, IRL) was performed with all dogs. It confirmed the presence of an increased proportion of granular lymphocytes, ranging from 12% to 66%, with a median of 53% (normal canine bone marrow contains less than 5% lymphocytes with rare granular lymphocytes). The lymphocytes had small nuclei and fairly sparse cytoplasm containing a few granules. In four patients, there was granulocytic hyperplasia with a left-shifted maturation sequence, leading to an elevated myeloid:erythroid ratio ranging from 6.6 to 26.0 (RI: 0.90–1.76) but with markedly reduced proportions of neutrophils. In two cases, there was granulocytic aplasia/hypoplasia, again with markedly reduced proportions of neutrophils and a low myeloid:erythroid ratio of 0.03–0.11 (RI: 0.9–1.76). Clonality analysis (PARR) for the B cell immunoglobulin heavy chain locus (IgH) and T cell receptor gamma (TCRγ) locus performed on bone marrow cytology slides (Stratagenem MX-3005P, Agilent Technologies, Stockport, UK) showed a clonal T cell arrangement in all four cases tested. Flow cytometry (Guava EasyCyte Flow Cytometry H2, Luminex Corporation, The Netherlands, NL) was performed on bone marrow in two cases and on peripheral blood in the patient with circulating LGLs, and it showed the neoplastic cells to be CD3+ CD45+ CD8+ lymphocytes, consistent with LGL T lymphocytes (Table 2).

### 3.2. Treatment

Following diagnosis, all patients were started on systemic chemotherapy. Five received chlorambucil (median dose 6 mg/m^2^ q24hr PO) and prednisolone (median dose 2 mg/kg q24hr PO), while one was treated with a vincristine/cyclophosphamide/prednisolone (COP)-based protocol. Due to the severe neutropaenia on presentation, five dogs were given the broad-spectrum antibiotic amoxicillin/clavulanic acid (median dose 18.4 mg/kg q12hr PO); one of these patients also received metronidazole (10 mg/kg q12hr PO). Antibiotic treatment was continued until normalisation of the neutropaenia. In three patients, prednisolone treatment was discontinued after 12, 14, and 29 weeks; these patients were continued on chlorambucil only. In two dogs, due to the lack of response to chlorambucil and prednisolone, different rescue treatments, including cyclosporine, melphalan, cyclophosphamide, vincristine, and mycophenolate, were used.

### 3.3. Outcomes

Normalisation of the neutrophil count was achieved in all six patients after a median of 15.1 weeks of treatment (range 1–19 weeks). The one patient treated with the COP-based protocol was euthanised 19 weeks after diagnosis due to uncontrolled gastrointestinal signs. One dog treated with the chlorambucil/prednisolone protocol as first-line and cyclosporine/cyclophosphamide/mycophenolate as rescue protocol was euthanised 46 weeks after diagnosis due to uncontrolled cytopaenia, melena, and copious epistaxis. Two dogs were lost to follow-up after 32 and 51 weeks. Two patients on treatment with chlorambucil and prednisolone were alive at the time of data collection, at a median follow-up of 674 days (679 and 721 days) at the time of data collection.

## 4. Discussion

LGL leukaemia in humans is an indolent disease which tends to affect elderly patients (median age at diagnosis of 66.5 years), with less than 15% of cases reported in people aged less than 50 years [1]. One-third of patients are asymptomatic at diagnosis [6,13]. Many patients develop immune-mediated neutropaenia. Common clinical features include aphthous ulceration, pyrexia (due to bacterial infection caused by neutropaenia), and rheumatoid arthritis. Splenomegaly is reported in one-quarter of patients, but hepatomegaly and peripheral lymphadenomegaly are reported to be extremely rare. Other immune-mediated conditions, such as lupus erythematosus, Sjogren’s syndrome, autoimmune thyroid disorders, inclusion body myositis, and coagulopathy, have rarely been reported [1]. Anaemia can be present [14], but the incidence of this finding has been demonstrated to be geographically variable. In one study, it was demonstrated that pure red cell aplasia (PRCA) secondary to LGL occurred approximately 10 times more frequently in Asian patients than in Western patients [15].

The typical form of chronic LGL lymphocytic leukaemia (LGL-CLL) in dogs is an indolent disease that represents 80% of all chronic leukaemias in dogs [7,16]. Cytologically, neoplastic lymphocytes contain a round nucleus with condensed chromatin and abundant cytoplasm containing azurophilic granules. This form of LGL-CLL arises in the spleen and is characterised by a circulating CD3+ CD5+ CD8+ C45+ MHCII+ lymphocytosis with mild or absent cytopaenias. Anaemia (75–86% cases) and thrombocytopaenia (15–45% cases) are mild, while neutropaenia has not been reported [7,8,12,16,17,18]. This disease usually affects middle-aged to older dogs, with a median age of 10 years (range 5–19 years) [12,16]. Most affected dogs are asymptomatic at the time of their diagnosis or, if present, the clinical signs are nonspecific [19]. Survival times are often prolonged (months to years). LGL-CLL needs to be distinguished from reactive lymphocytosis, which can be seen in dogs with a spectrum of infectious and/or inflammatory diseases, including ehrlichiosis [6,20,21].

In the variant of LGL-CLL described in this study, the lymphoid infiltrate in the bone marrow is fairly extensive (median 53% lymphocytes) but not sufficient to cause severe neutropaenia due to physical bone marrow crowding; therefore, cytopaenias are instead thought to be immune-mediated. In two cases, the bone marrow showed myeloid hypoplasia or aplasia, which suggests that the immune-mediated response had targeted the entire myeloid maturation sequence, including the early precursors. In the remaining four cases, there was myeloid hyperplasia but a marked reduction in neutrophils, suggesting that the immune-mediated response had targeted the neutrophil stage only. In this study, all affected dogs had severe neutropaenia on presentation, and some also had concurrent anaemia, thrombocytopaenia, and lymphopaenia. None of the dogs had peripheral lymphocytosis, but one patient showed an increased number of large granular lymphocytes, while the total number of peripheral lymphocytes remained within the reference range. The lack of peripheral lymphocytosis is a striking difference of this variant compared to the classic CLL, in which lymphocytosis is a key feature reported in all cases [16].

The diagnosis was made by combining bone marrow cytology, clonality testing (PARR), and flow cytometry. When performed, PARR analysis revealed T cell rearrangement, and flow cytometry revealed that the population of neoplastic lymphocytes was CD3+ CD5+ CD8+ T cells, as observed in humans. Screening for infectious diseases was performed in four cases. However, all cases came from a nonendemic area, and the possibility of an infectious cause in the non-tested cases was considered unlikely. An immune-mediated aetiology was therefore suspected to be the cause of the neutropaenia, given the bone marrow findings and the absence of an infectious disease or inflammatory focus, which could explain possible consumption. However, to confirm our hypothesis, immunological diagnostic tests, such as antineutrophil antibodies, should be performed.

In human medicine, patients with LGLL-related neutropaenia undergo a full diagnostic work-up to rule out other conditions, such as Epstein–Barr virus, cytomegalovirus, hepatitis B virus, hepatitis C virus, and human immunodeficiency virus [6]. Ultimately, a diagnosis of LGL leukaemia is made following evidence of an expanded clonal LGL population in the peripheral blood or bone marrow identified with clonality and flow cytometry. The neoplastic cells typically have a post-thymic phenotype, including TCR+, CD3+, CD4−, CD5dim, CD8+, CD16+, CD27−, CD28−, CD45R0−, CD45RA+, and CD57+. Bone marrow samples often show moderate to marked hypercellularity with interstitial lymphoid infiltration [2,22,23,24]. In general, in neutropaenic patients, a common characteristic observed on evaluation of the bone marrow is a decrease in granulocytic precursors along with left-shift myeloid maturation, which is reflected in an increase in immature myeloid precursors in the peripheral blood as well [2].

The pathological mechanisms underlying this condition are not fully understood. Three major mechanisms have been identified: humoral abnormalities, bone marrow infiltration/substitution, and, ultimately, cell-mediated cytotoxicity [2]. Humoral abnormalities include a heterogeneous spectrum of serological abnormalities, suggesting that inflammatory or autoimmune mechanisms can play an important role in the development of this condition [2]. These include the activation of survival pathways, including JAK/STAT and MAPK, interleukin 15, and somatic mutations of Stat3, Stat5b, and tumour necrosis factor alpha-induced protein 3 [6,10]. Additionally, reticulin fibrosis is usually increased in the bone marrow of LGLL patients and could play a role in the development of neutropaenia [25]. In terms of cell-mediated cytotoxic mechanisms, T and NK cells present different phenotypes based on the expression of antigens and receptors, many of which are members of the killer cell immunoglobulin-like receptor (KIR) family. In contrast, with the heterogenicity of KIR in the human population, LGLL shows a limited pattern of KIR characterised by a specific activated isoform, which is a typical feature of the leukaemic clone [26,27]. The activity of NK T cells is regulated by the activation and inhibition of KIR along with their ligands. A glitch between the activation of KIR combined with a lack of inhibitory signals is associated with neutropaenia and anaemia related to LGLL [2].

Since the Fas/Fas ligand apoptotic system is involved in regulating the survival of normal neutrophils, it has been hypothesised that LGLL-associated neutropaenia might be partially mediated by dysregulation of the Fas ligand, which is thought to be a possible trigger of the neutrophil apoptotic mechanism [28,29]. High levels of the Fas ligand (s-FasL) have been found in the sera of almost all patients with LGLL-associated neutropaenia [28,29].

Immunosuppressive treatment with chemotherapy drugs is the first-line treatment in humans with CLL [30,31]. Treatment is recommended when severe neutropaenia is detected (total count less than 0.5 × 10^9^/L) or in cases of moderate neutropaenia (total count more than 0.5 × 10^9^/L) that are associated with clinical signs related to secondary infections [6,32]. First-line treatments in human medicine include methotrexate or cyclophosphamide in combination with steroids (e.g., prednisolone). Rescue treatment with cyclosporine is recommended if both fail. However, a clinical response is seen in less than 35% of cases and usually takes 4 months [6].

A combination of chlorambucil and prednisolone is the first-line treatment in dogs with typical T and B cell CLL. Since CLL in dogs is usually an indolent condition, treatment is recommended for cases of advanced stage disease, including the presence of clinical signs (e.g., lethargy, anorexia, weight loss, etc.), infiltration of the spleen or lymph nodes, or in cases of anaemia or other cytopaenias. Severe peripheral lymphocytosis (>60,000 × 10^9^/L) or a rapidly increasing lymphocyte count are other important criteria to determine if a patient requires treatment [18].

In the present study, five dogs received chlorambucil and prednisolone as first-line treatment, and one received a COP-based protocol. In all patients, resolution of the neutropaenia was seen within 19 weeks of starting treatment. This is similar to what is reported in human medicine, in which a response is usually not expected before 16 weeks of treatment. However, a durable response was reported in only two patients. Due to severe neutropaenia, all patients were treated with broad spectrum systemic antibiotics. Survival was variable; only two patients were still alive at 679 and 721 days.

The limitations of this study are associated with its retrospective nature and the rarity of this condition. Other limitations include the limited number of cases collected, the lack of standardised investigations performed, and the different treatments used, which were dependent on the individual clinician.

## 5. Conclusions

A low percentage of dogs with LGL-CLL present clinical signs associated with severe neutropaenia, which is suspected to be immune-mediated, as well as other cytopaenias in some cases. Unlike the more common form of LGL-CLL, peripheral lymphocytosis is not observed in this rarer variant, and bone marrow biopsy is required to obtain a definitive diagnosis. Treatment with chlorambucil/prednisolone seems to be effective, but survival varies, and death related to cytopaenias can be expected. Further studies should be performed to better characterise this condition. LGL-CLL should be considered among the differential diagnosis in patients presenting with severe neutropaenia.

## Figures and Tables

**Table 1:** Haematology parameters of the patients included in the study.

Findings	Number of Patients (%)	Parameter	Median Value	Range	Reference
Anaemia	1 (16.6)	Haematocrit	0.32	0.32–0.44	0.37–0.55 L/L
Neutropaenia	6 (100)	Neutrophil count	0.07	0.02–0.76	3.0–11.5 × 10^9^/L
Thrombocytopaenia	3 (50)	Platelet count	66	50–102	200–500 × 10^9^/L
Lack of lymphocytosis	6 (100)	Lymphocytes	1.25	0.11–2.47	1.0–4.8 × 10^9^/L

**Table 2:** Bone marrow, PARR, and flow cytometry results for patients included in the study.

Test	Number of Patients (%)	Results
Bone marrow cytology	6 (100)	LGL T cell lymphocyte infiltration
PARR analysis on bone marrow	3 (50)	Clonal T cell arrangement
Flow cytometry on bone marrow aspirate	2 (33.3)	Identifying CD3+ and CD8+
Flow cytometry on peripheral blood	1 (16.6)	Identifying CD45+, CD3+, and CD8+ cytotoxic T lymphocytes

## Data Availability

The data presented in this study are available within the article. Raw data supporting this study are available from the corresponding author.

## References

[1] Moignet A., Lamy T. (2018). Latest advances in the diagnosis and treatment of large granular lymphocytic leukemia. Am. Soc. Oncol. Educ. Book.

[2] Calabretto G., Teramo A., Barilà G., Vicenzetto C., Gasparini V.R., Semenzato G., Zambello R. (2021). Neutropenia and Large granular Lymphocytes Leukaemia: From Pathogenesis to Therapeutic options. Cells.

[3] Loughran T. (1993). Clonal disease of large granular lymphocytes. Blood.

[4] Barilà G., Calabretto G., Teramo A., Vicenzetto C., Gasparini V.R., Semenzato G., Zambello R. (2019). T cell large granular leukemia and chronic NK lymphocytosis. Vest. Pract. Res. Clin. Haematol..

[5] Pontikoglou C., Kalpadakis C., Papadaki H.A. (2011). Pathophysiologic mehanisms and management of neutropenia associated with large granular lymphocytic leukaemia. Expert. Rev. Haemat..

[6] Lamy T., Moignet A., Loughran T.P. (2017). LGL leukaemia: From pathogenesis to treatment. Blood.

[7] Comazzi S., Gelain M.E., Martini V., Riondato F., Miniscalco B., Marconato L., Mortarino M. (2011). Immunophenotype Predict Survival Time in Dogs with Chronic Lymphocytic Leukaemia. J. Vet. Internal. Med..

[8] Leifer C.E. (1986). Canine lymphoma: Clinical considerations. Sem. Vet. Med. Surg. Small Anim..

[9] Museux K., Turinelli V., Rosemberg D., Rodriguez P. (2019). Chronic lymphopenia and neutropenia in a dog with large granular lymphocytes leukemia. Vet. Clin. Pathol..

[10] Elliot J., Villiers E. (2018). Indolent, T-cell, large granular lymphocytic leukaemia in a dog presenting with severe neutropenia and an absence of lymphocytosis. Open Vet. J..

[11] Adam F., Villiers E., Watson S., Coyne K., Blackwood L. (2009). Clinical Pathological epidemiological assessment and morphologically and immunologically confirmed canine leukaemia. Vet. Comp. Onc..

[12] Burks E.J., Loughran T.P. (2005). Perspectives in the treatment of LGL leukemia. Leuk. Res..

[13] Lamy T., Loughran T.P. (2011). How I treat LGL leukaemia. Blood.

[14] Kwong Y.L., Wong K.F. (1998). Association of pure red cell aplasia with T large granular lymphocytes leukaemia. J. Clin. Pathol..

[15] Kwong Y.L. (2012). Pathogenesis and treatment of leukemia: An Asian perspective. Exp. Opin. Ther. Targets.

[16] Tasca S., Carli E., Caldin M., Menegazzo L., Furlanello T., Gallego L.S. (2009). Hematologic abnormalities and flow cytometric immunophenotyping results in dogs with hematopoietic neoplasia: 210 cases (2002–2006). Vet. Clin. Pathol..

[17] Vernau W., Moore P.F. (1999). An immunophenotypic study of canine leukaemias and preliminary assessment of clonality by polymerase chain reaction. Vet. Immunol. Immunopathol..

[18] Workman H.C., Vernau W. (2003). Chronic lymphocytic leukaemia in dogs and cats: The veterinary perspective. Vet. Clin. Small Anim..

[19] Leifer C.E., Matus R.E. (1986). Chronic lymphocytic leukemia in the dog: 22 cases (1974–1984). J. Am. Vet. Assoc..

[20] McDonough S.P., Moore P.F. (2000). Clinical, hematologic, and immunophenotypic characterization of canine large granular lymphocytosis. Vet Pathol..

[21] Heeb H.L., Wilkerson M.J., Chun R., Ganta R.R. (2003). Large Granular Lymphocytosis, Lymphocute Subset inversion, Thrombocytopenia, Dysproteinemia, and Positive Ehrlichia Serology in a Dog. J. Am. Anim. Hops. Assoc..

[22] Picker L.J., Kadin E., Furst A., Robinson H. (1987). Immunoarchitecture of the bone marrow in neutropenia: Increased HNK-1 + cells define a subset of neutropenic patients. Am. J. Haematol..

[23] Osuji N., Randen U., Matutes E., Tjonnfjord G., Catocsky D., Wotherspoon A. (2007). Characteristic appearances of the bone marrow T-cell large granular lymphocytes leukaemia. Histopathology.

[24] Mailloux A., Zhang L., Moscinski L., Bennett J., Yang L., Yoder S., Epling-Burnette P. (2013). Pathophysiology of cytopenias in an autoimmune lymphoproliferative disease linked to bone marrow fibrosis (P3165). J. Immunol..

[25] Mailloux A.W., Zhang L., Moscinski L., Bennett J.M., Yang L., Yoder S.J., Epling-Burnette P.K. (2013). Fibrosis and Subsequent Cytopenias are Associated with bfGf-deficient Pluripotent Mesenchymal Stromal Cells in Large Granular Lymphocytes Leukemia. J. Immunol..

[26] Zambello R., Falco M., Chiesa M.D., Trentin L., Carollo D., Castriconi R., Vitale M. (2003). Expression and function of KIR and natural cytotoxicity receptors in NK-type lymphoproliferative diseases of granular lymphocytes. Blood.

[27] Scquizzato E., Teramo A., Miorin M., Facco M., Piazza F., Noventa F., Trentin L., Agostini C., Zambello R. (2007). Genotypic evaluation of killer immunoglobulin-like receptors in NK-type lymphoproliferative disease of granular lymphocytes. Leukemia.

[28] Liu J.H., Wei S., Lamy T., Epling-Burnette P.K., Starkebaum G., Djeu J.Y., Loughran T.P. (2000). Chronic neutropenia mediated by Fas ligand. Blood.

[29] Liu J.H., Lamy S., Li Y., Epling-Burnette P.K., Djeu J.Y., Loughran T.P. (2002). Blockage of Fas-dependent apoptosis by soluble Fas in LGL leukemia. Blood.

[30] Moignet A., Hasanali Z., Zambello R., Pavan L., Bareau B., Tournilhac O., Lamy T. (2014). Cyclophosphamide as fist-line therapy in LGL leukemia. Leukemia.

[31] Loughran T.P., Zickl L., Olson T.L., Wang V., Zhang D., Rajala H.L., Tallman M.S. (2015). Immunosuppressive therapy of LGL leukemia:prospective multicenter phase II study by the Eastern Cooperative Oncology Group (E5998). Leukemia.

[32] Lustberg M. (2012). Management of Neutropenia in Cancer Patients. Clinc. Adv. Haematol. Oncol..

